# Flavonoid-rich dietary patterns and incident hospital-recorded Parkinson's disease: a prospective UK Biobank study

**DOI:** 10.3389/fnut.2026.1893517

**Published:** 2026-08-10

**Authors:** Wen Yang, Zhetian Chen, Yuanyuan Zhang, Cong Peng, Bin Chen

**Affiliations:** 1 Department of Pathology, Affiliated Hospital of Guizhou Medical University, Yunyan, Guiyang, Guizhou, China; 2 Department of Pathology, Guizhou Medical University, Yunyan, Guiyang, Guizhou, China; 3 Department of Neurology, The Second Affiliated Hospital of Hainan Medical University, Haikou, Hainan, China; 4 Department of Otolaryngology-Head and Neck Surgery, Renmin Hospital of Wuhan University, Wuchang, Wuhan, Hubei, China; 5 Research Institute of Otolaryngology-Head and Neck Surgery, Renmin Hospital of Wuhan University, Wuchang, Wuhan, Hubei, China; 6 Department of Otolaryngology, Guizhou Provincial People's Hospital, Nanming, Guiyang, Guizhou, China

**Keywords:** flavonoid diet score, Parkinson's disease, proteomics, single-nucleus transcriptomics, UK Biobank

## Abstract

**Introduction:**

Evidence linking flavonoid-rich dietary patterns to the risk of Parkinson's disease (PD) remains limited and inconsistent, and molecular clues to this association remain scarce. This study examined the association between a flavonoid diet score (FDS) and incident hospital-recorded PD and explored related plasma proteomic and cellular expression patterns.

**Methods:**

In this prospective analysis of 123,655 UK Biobank participants, we used multivariable Cox proportional hazards models to estimate hazard ratios (HRs) and 95% confidence intervals (CIs) for the association between FDS and incident hospital-recorded PD. In an exploratory analysis, we integrated a plasma proteomic screen with publicly available single-cell and single-nucleus transcriptomic data to provide preliminary molecular and cellular context.

**Results:**

During follow-up, 796 incident hospital-recorded PD cases were identified. Higher FDS was associated with a lower risk of incident hospital-recorded PD per 1-standard deviation (SD) increase (HR, 0.93; 95% CI, 0.86–1.00), and the risk was lower in the highest than in the lowest FDS quartile (HR, 0.75; 95% CI, 0.61–0.93). In decomposition analyses, no individual food source or flavonoid subclass remained associated with incident hospital-recorded PD after multiple-testing correction. The inverse association was directionally consistent across most sensitivity analyses and in 1–5-year lag analyses, but the evidence became borderline after adjustment for coffee intake (HR, 0.93; 95% CI, 0.86–1.00) and was no longer statistically supported in 6–10-year lag analyses. In exploratory omics analyses, the proteomic screen prioritized a direction-compatible signature comprising 24 proteins, including proteins related to immune and adhesion processes, while single-cell and single-nucleus transcriptomic data provided peripheral immune and glial-vascular cellular context.

**Discussion:**

In this prospective cohort, higher FDS was modestly associated with a lower incidence of hospital-recorded PD. Residual confounding and reverse causation remain possible, and the proteomic and transcriptomic findings should be considered hypothesis-generating.

## 1 Introduction

Parkinson's disease (PD) is a common neurodegenerative disorder with a rising global burden, and its progressive motor and non-motor symptoms markedly impair quality of life and contribute to disability and mortality (1–4). Effective disease-modifying therapies are still lacking, and clinical management remains largely symptomatic (5). Against this background, diet, as a modifiable lifestyle factor, has received growing attention in PD prevention research. Prospective studies have shown that several healthy dietary patterns, including the Mediterranean, Mediterranean-DASH Intervention for Neurodegenerative Delay (MIND), and plant-forward diets, are associated with lower PD risk (6–8). Building on this, exploring further dietary patterns in relation to PD may generate additional lifestyle-related hypotheses for future PD risk research.

Polyphenols are a class of bioactive compounds widely present in plant-based foods, and flavonoids are one of the major subclasses of dietary polyphenols, mainly derived from foods such as tea, berries, citrus fruits, apples, and cocoa (9, 10). Recent experimental studies and reviews suggest that flavonoids may exert neuroprotective effects by reducing oxidative stress, modulating neuroinflammation, and improving mitochondrial function, processes implicated in PD pathogenesis (11, 12).

However, important gaps remain in the existing evidence. An earlier prospective study suggested that higher flavonoid intake, particularly anthocyanins, might be associated with reduced risk of PD, whereas updated long-term cohort analyses did not identify a clear inverse association for total flavonoid intake or its subclasses (13, 14). Moreover, most previous studies have focused on individual flavonoids or total flavonoid intake rather than flavonoid-related dietary patterns. In addition, previous diet–PD research has been largely dominated by observational epidemiological studies, with molecular and cellular contextual evidence remaining comparatively limited (15–18).

To address these gaps, the present study constructed a flavonoid diet score (FDS) in the UK Biobank prospective cohort and systematically examined its association with risk of incident hospital-recorded PD. In addition, an exploratory screen of baseline plasma proteomic data was used to identify proteins associated with both FDS and incident hospital-recorded PD, and publicly available peripheral blood mononuclear cell (PBMC) single-cell and substantia nigra single-nucleus transcriptomic datasets were used to interpret their cellular context, thereby offering molecular and cellular clues to this association.

## 2 Materials and methods

### 2.1 Study design and data sources

This prospective cohort analysis was based on the UK Biobank, a population-based cohort with baseline questionnaires, physical measurements, biospecimens, and long-term follow-up via linkage to hospital inpatient and death registry records (19). The primary analysis evaluated the association between FDS and incident hospital-recorded PD. Additional analyses integrated baseline plasma proteomic data and publicly available single-cell and single-nucleus transcriptomic datasets to explore potential biological contexts underlying the observed association. External transcriptomic resources included a peripheral blood mononuclear cell (PBMC) single-cell RNA sequencing (scRNA-seq) dataset from the Gene Expression Omnibus (GEO; accession GSE223138) comprising six participants (two with early-stage PD, two with advanced-stage PD, and two matched controls), and a postmortem substantia nigra pars compacta single-nucleus RNA sequencing (snRNA-seq) dataset (accession GSE243639) comprising 29 donors (15 with sporadic PD and 14 controls) (20, 21).

### 2.2 Study population

**Figure 1:**
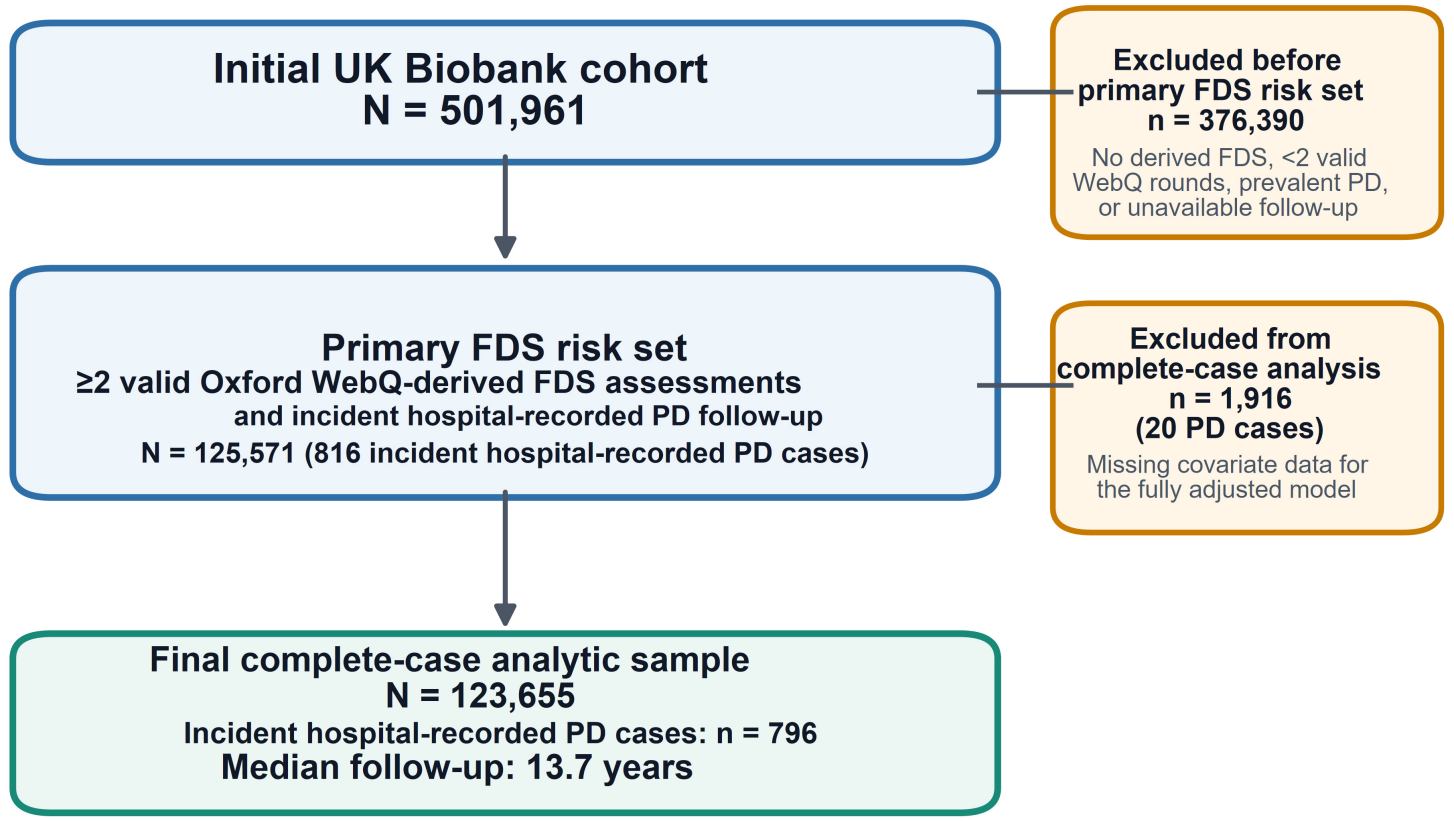
Flowchart of participant selection for the prospective analysis. From 501,961 UK Biobank participants, 123,655 participants with available FDS data, incident hospital-recorded PD follow-up, and complete covariate data were included in the final analysis. During a median follow-up of 13.7 years, 796 incident hospital-recorded PD cases were identified. FDS, flavonoid diet score; PD, Parkinson's disease.

Participants were eligible if they were free of PD at baseline, had at least two valid Oxford WebQ assessments for FDS calculation, and had available follow-up data for incident hospital-recorded PD ascertainment. Among eligible participants, 125,571 individuals were identified. A complete-case approach was applied for the primary analysis, and 1,916 participants with missing data for one or more prespecified covariates in the fully adjusted model were excluded, resulting in a final analytic cohort of 123,655 participants. Follow-up extended from the baseline assessment date to the first occurrence of hospital-recorded PD, death, or administrative censoring on 31 March 2023, whichever occurred first. The participant selection process is illustrated in Figure 1.

### 2.3 Flavonoid diet score (FDS)

The FDS summarizes habitual intake of 11 flavonoid-rich food groups captured by repeated Oxford WebQ 24-h dietary recalls, including black tea, green tea, red wine, apples, berries, grapes, oranges, grapefruit, sweet peppers, onions, and dark chocolate. Intake of each food group was quantified as daily servings and combined into an overall FDS according to a previously published scoring scheme (22). Each component contributed positively to the total score, with higher intake of these flavonoid-rich food groups corresponding to higher FDS values; no component was assigned a negative contribution. Food-group definitions, field IDs, response-code recoding, and units are provided in Supplementary Table 1. For each participant, the mean of available round-specific FDS values was used as the exposure. The primary analysis included participants with at least two valid Oxford WebQ assessments, with alternative thresholds of at least one, three, and four assessments evaluated in sensitivity analyses. For continuous analyses, FDS was standardized as a *Z*-score within the primary analytic sample, and hazard ratios (HRs) were estimated per 1-standard deviation (SD) increase. For categorical analyses, participants were categorized into quartiles according to their mean FDS distribution.

### 2.4 Ascertainment of incident hospital-recorded PD

Incident hospital-recorded PD was identified through UK Biobank linkage to hospital inpatient data and death registries using International Classification of Diseases, Tenth Revision (ICD-10) codes (Supplementary Table 2). Participants with hospital-recorded PD at baseline were excluded, and the event date was defined as the earliest hospital-recorded PD diagnosis during follow-up.

### 2.5 Covariates

Covariates were selected *a priori*: age, sex, ethnicity, educational attainment, Townsend deprivation index (TDI), body mass index (BMI), smoking status, alcohol consumption, physical activity, sleep pattern, total energy intake, and number of long-term morbidities (Supplementary Tables 2, 3). Total energy intake was derived from repeated Oxford WebQ rounds; values outside 500–4,500 kcal/day were set to missing, and remaining values were averaged across rounds. Additional covariates, including Mediterranean diet adherence, habitual coffee intake, PD polygenic risk score (PRS), and self-rated overall health, were evaluated in sensitivity analyses. The Mediterranean diet adherence proxy is described in Supplementary Table 4, while the derivations for habitual coffee intake and self-rated health are provided in Supplementary Table 2.

### 2.6 Plasma proteomics

UK Biobank Olink Explore plasma proteomics provided Normalized Protein eXpression (NPX) measurements for 2,923 proteins in approximately 54,219 participants, quantified by proximity extension assay under standardized preanalytical conditions (23). After linkage to the primary analytic cohort, 13,076 participants with 163 incident hospital-recorded PD events had baseline Olink measurements and were included in the proteomics subset (Supplementary Table 5). Proteins with >20% missing NPX values in this subset were excluded, leaving 2,908 quality-controlled proteins for screening. Residual missing NPX values were not imputed; protein-specific models were fitted using available observations for each protein (Supplementary Table 6). NPX values were Z-standardized before analysis to allow effect estimates to be interpreted per 1-SD higher protein level.

### 2.7 Single-cell and single-nucleus transcriptomic analysis

External PBMC scRNA-seq (GEO: GSE223138) and substantia nigra pars compacta snRNA-seq (GEO: GSE243639) datasets were processed using standardized workflows. For quality control, cells/nuclei with fewer than 200/300 or more than 6,000 detected genes, or mitochondrial transcript proportion >20%, were excluded for PBMC and substantia nigra datasets, respectively; genes detected in fewer than three cells/nuclei were removed. After quality control, 63,671 PBMC cells and 165,488 substantia nigra nuclei were retained. Both datasets were normalized, log-transformed, and integrated using Harmony with sample identity as the batch factor. Dimensionality reduction, clustering, and cell-type annotation were performed using principal component analysis (PCA), uniform manifold approximation and projection (UMAP), Leiden clustering, and canonical marker genes. Area under the curve-based gene set activity scoring (AUCell) was used to characterize protective-aligned and risk-aligned signature activity across cell populations. Detectable signature genes were summarized by cell type, and CellPhoneDB was applied for exploratory annotation of signature-related ligand–receptor and adhesion-associated interaction patterns.

### 2.8 Statistical analysis

Baseline characteristics were summarized according to quartiles of the FDS. Continuous variables were compared using analysis of variance or Kruskal–Wallis tests, as appropriate, and categorical variables were compared using chi-square tests. Associations between FDS and incident hospital-recorded PD were evaluated using Cox proportional hazards models. FDS was modeled both as a continuous variable per 1-SD increase and as quartiles, with the lowest quartile as the reference group. Sequential models were fitted, including an unadjusted model, a demographic-adjusted model adjusted for age, sex, and ethnicity, and a fully adjusted model further adjusted for education, TDI, BMI, smoking status, alcohol consumption, physical activity, sleep pattern, total energy intake, and number of long-term conditions. Linear trend across FDS quartiles was assessed by modeling the median value of each quartile as a continuous variable. The proportional hazards assumption was assessed using Schoenfeld residuals. Effect estimates are reported as HRs with 95% confidence intervals (CIs).

To characterize the stability of repeated dietary assessment, the distribution and temporal spacing of valid Oxford WebQ rounds were summarized, and FDS reproducibility across assessment rounds was evaluated using pairwise Pearson and Spearman correlations, Cronbach's alpha, and the intraclass correlation coefficient (ICC). The primary analysis was restricted to participants with at least two valid Oxford WebQ assessments. Sensitivity analyses were conducted using alternative thresholds of at least one, at least three, and at least four valid WebQ assessments. Additional robustness analyses included energy-residual FDS modeling, exclusion of extreme FDS values, lag analyses using progressively extended exclusion windows (1–10 years), Fine–Gray competing-risk models treating death as a competing event, inverse probability of complete-case weighting (IPCCW), and propensity-score weighted analyses comparing the highest FDS quartile with the remaining quartiles using inverse probability treatment weighting (IPTW) and overlap weighting. Additional adjustment analyses included Mediterranean diet adherence, PD polygenic risk score, habitual coffee intake, and self-rated overall health.

Dose-response patterns were further examined by categorizing FDS into quintiles and deciles, with the lowest category as the reference group. Trend tests were performed by assigning the median FDS value within each category as a continuous variable. Non-linearity was evaluated using restricted cubic splines with knots at the 5th, 35th, 65th, and 95th percentiles. Exploratory fully adjusted Cox models examined individual FDS food sources and food-based flavonoid subclass proxies. Multiple testing within each analysis set was controlled using the Benjamini–Hochberg false discovery rate (FDR) procedure.

Proteomic analyses were performed among participants with available baseline Olink plasma proteomic data and complete covariate information. For each protein, multivariable linear regression was used to estimate the association between FDS and standardized NPX levels, and Cox proportional hazards models were used to evaluate the association between standardized protein levels and incident hospital-recorded PD. Both analyses used the fully adjusted covariate set. A symmetric screening strategy was applied by intersecting proteins associated with FDS and incident hospital-recorded PD at nominal *p* < 0.05. Proteins with directions compatible with the inverse FDS–PD association were retained as a direction-compatible protein signature and classified as protective-aligned or risk-aligned according to the direction of associations. A stricter sensitivity analysis requiring FDR significance in both screening steps was additionally performed.

Proteins within the direction-compatible signature were functionally annotated using over-representation analyses based on Gene Ontology Biological Process and Kyoto Encyclopedia of Genes and Genomes (KEGG) pathway libraries, with multiple-testing-adjusted *p*-values reported. Protein–protein interaction patterns were evaluated using the Search Tool for the Retrieval of Interacting Genes/Proteins (STRING), and network connectivity was summarized using degree-based measures.

For single-cell and single-nucleus transcriptomic contextualization, direction-defined proteomic signatures were scored across annotated cell populations using AUCell. Expression patterns of detectable signature genes were summarized by cell type. CellPhoneDB was applied for exploratory annotation of signature-related ligand–receptor and adhesion-associated interaction patterns at the broad cell-type level.

All tests were two-sided. A nominal *p* < 0.05 was considered statistically significant unless otherwise specified. False discovery rate adjustment was used for multiple-testing summaries. Analyses were conducted using R (4.6.0) and Python (3.12.13), and the main software packages and versions are listed in Supplementary Table 7.

## 3 Results

### 3.1 Study population and baseline characteristics

A total of 123,655 participants with at least two valid Oxford WebQ assessments were included in the primary analysis, with 796 incident hospital-recorded PD cases documented during follow-up. Participants completed a median of 3 valid Oxford WebQ assessments, with a mean interval of 247.7 days between consecutive dated assessments. Repeated FDS measurements showed moderate reproducibility, with median pairwise Pearson and Spearman correlations of 0.633 and 0.638, respectively, a Cronbach's alpha of 0.892, and an intraclass correlation coefficient of 0.630 (Supplementary Table 8). Participants were categorized into four quartiles according to the FDS distribution. Compared with participants in the lowest FDS quartile, those in the highest quartile were older, more likely to be of White ethnicity, less deprived (lower TDI), and had a more favorable lifestyle profile, including lower obesity prevalence and higher physical activity. Smoking status, alcohol consumption, sleep pattern, long-term morbidity burden, and total energy intake also differed across quartiles (Table 1). Compared with participants without a derived FDS score or with fewer than two valid Oxford WebQ assessments, participants with at least two valid WebQ-derived FDS assessments had a higher proportion of White ethnicity, lower socioeconomic deprivation, lower obesity prevalence, lower current smoking prevalence, and a lower crude hospital-recorded PD incidence rate (Supplementary Table 9).

**Table 1:** Baseline characteristics of participants included in the incident hospital-recorded PD analysis according to quartiles of FDS.

Variable	Overall (*n* = 123,655)	Q1 [0.00, 2.25] (*n* = 30,914)	Q2 (2.25, 3.75] (*n* = 30,914)	Q3 (3.75, 5.25] (*n* = 30,914)	Q4 (5.25, 18.75] (*n* = 30,913)	*p-*value
Age, years	56.13 (7.82)	54.75 (8.08)	56.03 (7.88)	56.67 (7.69)	57.07 (7.41)	< 0.001
Sex, *n* (%)						0.466
Female	69,097 (55.9)	17,328 (56.1)	17,305 (56.0)	17,310 (56.0)	17,154 (55.5)	
Male	54,558 (44.1)	13,586 (43.9)	13,609 (44.0)	13,604 (44.0)	13,759 (44.5)	
Ethnicity, *n* (%)						< 0.001
White	119,861 (96.9)	29,547 (95.6)	29,795 (96.4)	30,115 (97.4)	30,404 (98.4)	
Non-white	3,794 (3.1)	1,367 (4.4)	1,119 (3.6)	799 (2.6)	509 (1.6)	
Education, *n* (%)						0.856
Unknown	23,446 (19.0)	5,841 (18.9)	5,895 (19.1)	5,854 (18.9)	5,856 (18.9)	
College	39,933 (32.3)	10,058 (32.5)	9,975 (32.3)	9,893 (32.0)	10,007 (32.4)	
Other	60,276 (48.7)	15,015 (48.6)	15,044 (48.7)	15,167 (49.1)	15,050 (48.7)	
TDI, median (IQR)	−2.37 (−3.77 to −0.04)	−2.13 (−3.65 to 0.43)	−2.34 (−3.75 to 0.00)	−2.48 (−3.82 to −0.24)	−2.51 (−3.83 to −0.38)	< 0.001
BMI, *n* (%)						< 0.001
Underweight	707 (0.6)	166 (0.5)	196 (0.6)	160 (0.5)	185 (0.6)	
Normal	48,399 (39.1)	11,101 (35.9)	12,314 (39.8)	12,568 (40.7)	12,416 (40.2)	
Overweight	50,479 (40.8)	12,324 (39.9)	12,609 (40.8)	12,719 (41.1)	12,827 (41.5)	
Obese	24,070 (19.5)	7,323 (23.7)	5,795 (18.7)	5,467 (17.7)	5,485 (17.7)	
Smoking, *n* (%)						< 0.001
Never	70,802 (57.3)	17,838 (57.7)	17,828 (57.7)	17,929 (58.0)	17,207 (55.7)	
Previous	44,319 (35.8)	10,398 (33.6)	11,101 (35.9)	11,167 (36.1)	11,653 (37.7)	
Current	8,534 (6.9)	2,678 (8.7)	1,985 (6.4)	1,818 (5.9)	2,053 (6.6)	
Alcohol, *n* (%)						< 0.001
Never	3,498 (2.8)	1,144 (3.7)	820 (2.7)	724 (2.3)	810 (2.6)	
Previous	3,516 (2.8)	1,127 (3.6)	792 (2.6)	723 (2.3)	874 (2.8)	
Current	116,641 (94.3)	28,643 (92.7)	29,302 (94.8)	29,467 (95.3)	29,229 (94.6)	
Physical activity, *n* (%)						< 0.001
No	48,024 (38.8)	12,963 (41.9)	12,082 (39.1)	11,860 (38.4)	11,119 (36.0)	
Yes	75,631 (61.2)	17,951 (58.1)	18,832 (60.9)	19,054 (61.6)	19,794 (64.0)	
Sleep pattern, *n* (%)						< 0.001
Healthy	70,254 (56.8)	16,814 (54.4)	17,653 (57.1)	17,926 (58.0)	17,861 (57.8)	
Moderate	50,060 (40.5)	13,118 (42.4)	12,454 (40.3)	12,260 (39.7)	12,228 (39.6)	
Poor	3,341 (2.7)	982 (3.2)	807 (2.6)	728 (2.4)	824 (2.7)	
No. of long-term morbidities, *n* (%)						< 0.001
0	65,160 (52.7)	16,131 (52.2)	16,627 (53.8)	16,330 (52.8)	16,072 (52.0)	
1	39,910 (32.3)	9,964 (32.2)	9,861 (31.9)	9,974 (32.3)	10,111 (32.7)	
2	14,137 (11.4)	3,634 (11.8)	3,385 (10.9)	3,530 (11.4)	3,588 (11.6)	
≥3	4,448 (3.6)	1,185 (3.8)	1,041 (3.4)	1,080 (3.5)	1,142 (3.7)	
FDS, median (IQR)	3.75 (2.25 to 5.25)	1.25 (0.67 to 1.75)	3.00 (2.62 to 3.38)	4.50 (4.06 to 4.88)	6.30 (5.75 to 7.08)	< 0.001
Total energy intake, mean (SD), kcal/day	2,092.09 (499.34)	2,014.50 (505.08)	2,068.25 (484.68)	2,111.62 (488.63)	2,174.00 (504.89)	< 0.001

Continuous variables were compared using analysis of variance or Kruskal–Wallis tests as appropriate. FDS quartiles were generated using rank-based four-group classification; displayed FDS ranges are descriptive because tied FDS values occurred at quartile boundaries. Categorical variables were compared using χ^2^ tests.

BMI, body mass index; FDS, flavonoid diet score; IQR, interquartile range; PD, Parkinson's disease; SD, standard deviation; TDI, Townsend deprivation index.

### 3.2 Association between FDS and incident hospital-recorded PD

**Figure 2:**
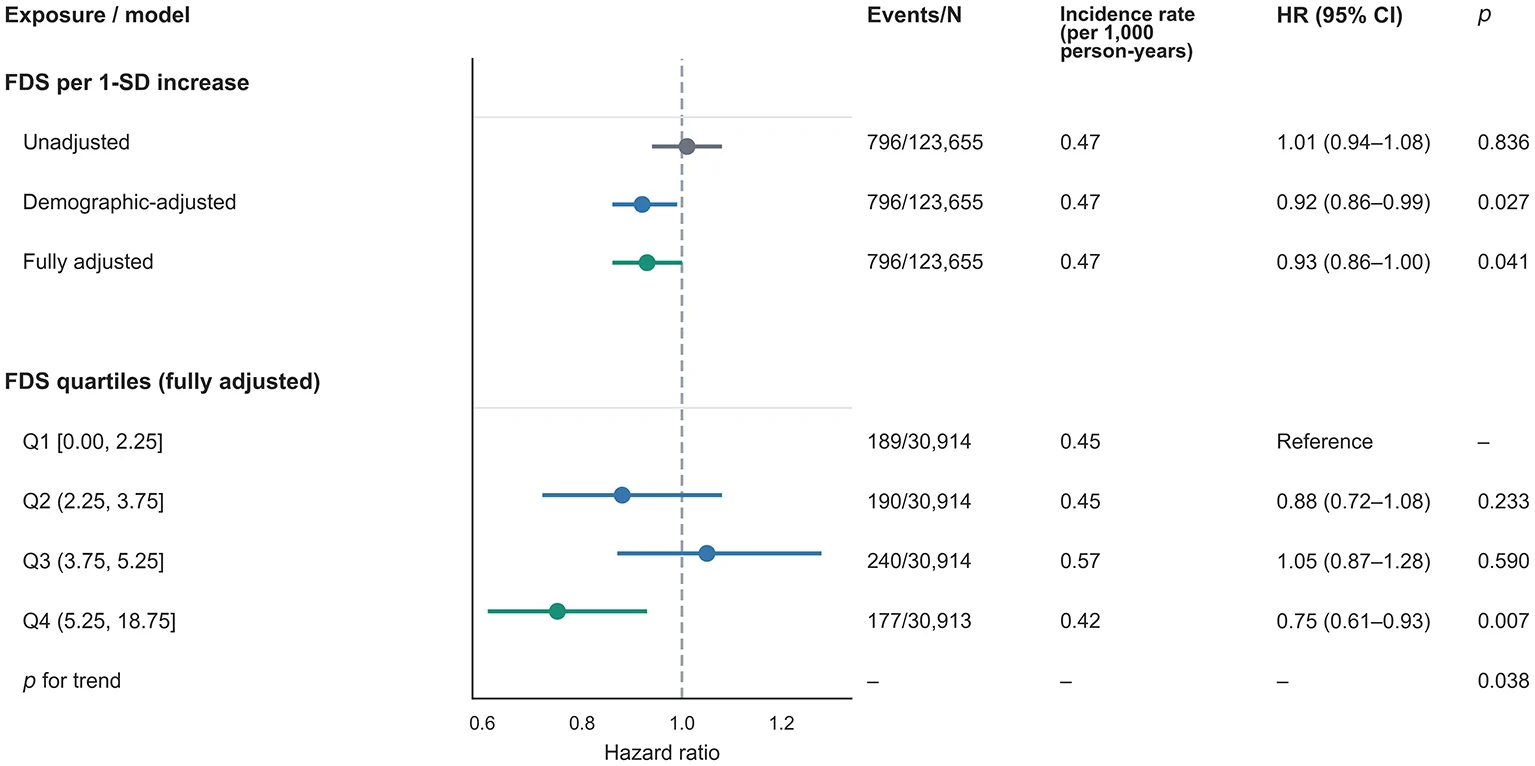
Association between the FDS and incident hospital-recorded PD. The demographic-adjusted model included age, sex, and ethnicity; the fully adjusted model additionally included socioeconomic, lifestyle, energy intake, sleep, and morbidity covariates. CI, confidence interval; FDS, flavonoid diet score; HR, hazard ratio; PD, Parkinson's disease.

In the fully adjusted model, each 1-SD increase in FDS was associated with an HR of 0.93 (95% *CI*, 0.86–1.00). In quartile analyses, the inverse association was mainly observed in the highest FDS quartile (Q4 vs. Q1: *HR*, 0.75; 95% *CI*, 0.61–0.93), with no strictly monotonic gradient across intermediate quartiles (Figure 2). Schoenfeld residual tests showed no evidence of violation for the FDS term (*p* = 0.845) or the overall model (*p* = 0.166; Supplementary Table 10). Additional categorical analyses further characterized the dose-response pattern. The highest quintile showed a lower hospital-recorded PD incidence estimate compared with the lowest quintile (Q5 vs. Q1: *HR*, 0.69; 95% *CI*, 0.55–0.87), and similar estimates were observed in the highest decile (D10 vs. D1: *HR*, 0.71; 95% *CI*, 0.51–0.98). Trend analyses across quintiles and deciles are presented in Supplementary Table 11. Restricted cubic spline analysis did not indicate evidence of a non-linear association between FDS and incident hospital-recorded PD (Supplementary Figure 1).

Exploratory component-level analyses did not identify an individual food source or subclass-rich proxy that remained associated with incident hospital-recorded PD after FDR correction. The total flavonoid-rich proxy showed a directionally similar estimate, whereas individual subclass proxies did not show stronger associations (Supplementary Tables 12, 13).

Sensitivity analyses using alternative WebQ assessment thresholds showed generally consistent inverse associations. Across ≥1, ≥2, ≥3, and ≥4 valid WebQ thresholds, continuous FDS models showed similar estimates (HR range, 0.85–0.93), although precision decreased at higher WebQ thresholds. The highest FDS quartile remained associated with lower hospital-recorded PD incidence across thresholds (Q4 vs. Q1 HR range, 0.61–0.76; Supplementary Table 14). Lag analyses showed directionally similar estimates after excluding incident hospital-recorded PD cases occurring within the first 1–5 years of follow-up (HR range, 0.92–0.93), whereas estimates were attenuated after longer 6–10-year exclusion windows (HR range, 0.94–0.95; Supplementary Table 15).

Other robustness analyses yielded directionally similar results. The association was largely unchanged after energy-residual FDS modeling (*HR*, 0.93; 95% *CI*, 0.86–1.00), exclusion of extreme FDS values (*HR*, 0.91; 95% *CI*, 0.84–0.98), and additional adjustment for Mediterranean diet adherence, PD polygenic risk score, or self-rated overall health, with HRs remaining approximately 0.92–0.93. Additional adjustment for coffee intake yielded a similar inverse point estimate, although the confidence interval extended to the null and the association was no longer statistically significant at the conventional threshold (*HR*, 0.93; 95% *CI*, 0.86–1.00). A Fine–Gray competing-risk model yielded a similar subdistribution hazard ratio (SHR, 0.93; 95% *CI*, 0.87–1.00), and complete-case weighting produced nearly identical results (*HR*, 0.93; 95% *CI*, 0.86–1.00). In high-FDS group comparisons, both IPTW and overlap weighting supported lower risk of incident hospital-recorded PD among participants in the highest FDS quartile vs. Q1–Q3 (IPTW: *HR*, 0.73; 95% *CI*, 0.62–0.87; overlap weighting: *HR*, 0.75; 95% *CI*, 0.63–0.88; Supplementary Table 16).

### 3.3 Plasma proteomic signature associated with FDS and incident hospital-recorded PD

**Figure 3:**
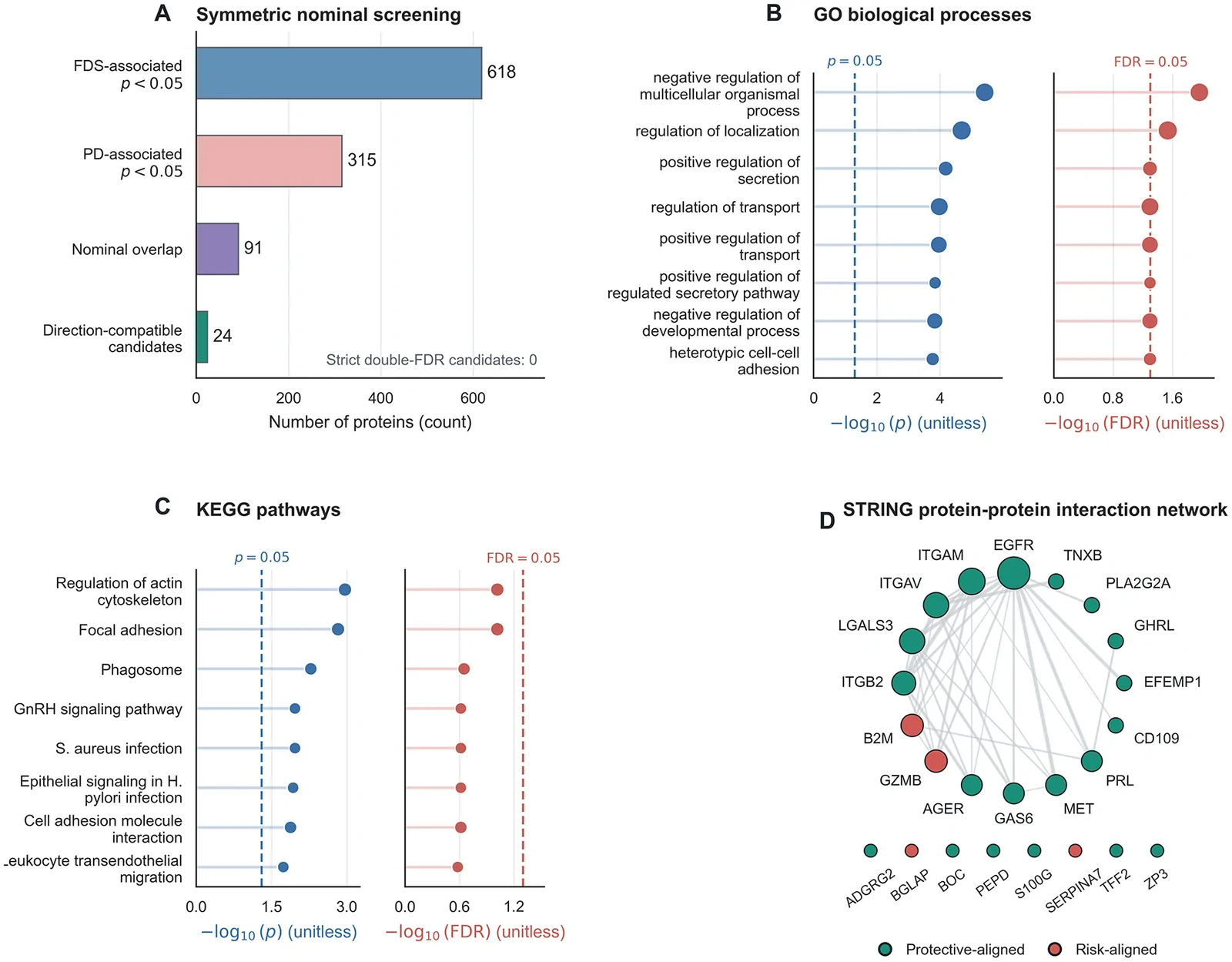
Direction-compatible proteomic prioritization and functional annotation. **(A)** Symmetric proteomic screening strategy. **(B, C)** GO biological process and KEGG pathway enrichment analyses of the 24 proteins comprising the exploratory direction-compatible protein signature. **(D)** STRING protein–protein interaction network constructed from this signature. Protein sets associated with FDS and incident hospital-recorded PD at a nominal significance threshold of *p* < 0.05 were intersected, and those with effect directions consistent with the inverse FDS–PD association were retained as direction-compatible proteins. FDR, false discovery rate; FDS, flavonoid diet score; GO, gene ontology; KEGG, Kyoto encyclopedia of genes and genomes; PD, Parkinson's disease; STRING, search tool for the retrieval of interacting genes/proteins.

Among participants with available baseline Olink proteomic data (*n* = 13,076; 163 incident hospital-recorded PD events; 2,908 proteins), a symmetric nominal screening approach yielded 91 proteins associated with both FDS and incident hospital-recorded PD, of which 24 showed directions compatible with the inverse FDS–PD association (20 protective-aligned and 4 risk-aligned proteins). No individual protein remained significant after FDR correction in both screening steps (Figure 3A; Supplementary Table 17). Functional enrichment was weak, with only two broad Gene Ontology terms passing *FDR* < 0.05 and no significant KEGG pathway (lowest *FDR* = 0.096). The nominally enriched terms related broadly to adhesion, transport, and immune processes (Figures 3B, C; Supplementary Tables 18, 19). STRING analysis demonstrated network connectivity among 16 of the 24 proteins through 36 edges, with EGFR, ITGAM, ITGAV, LGALS3, ITGB2, GZMB, B2M, and GAS6 showing relatively higher network connectivity within the exploratory protein-signature network (Figure 3D; Supplementary Tables 20, 21).

### 3.4 Cellular contextualization of the plasma proteomic signature

**Figure 4:**
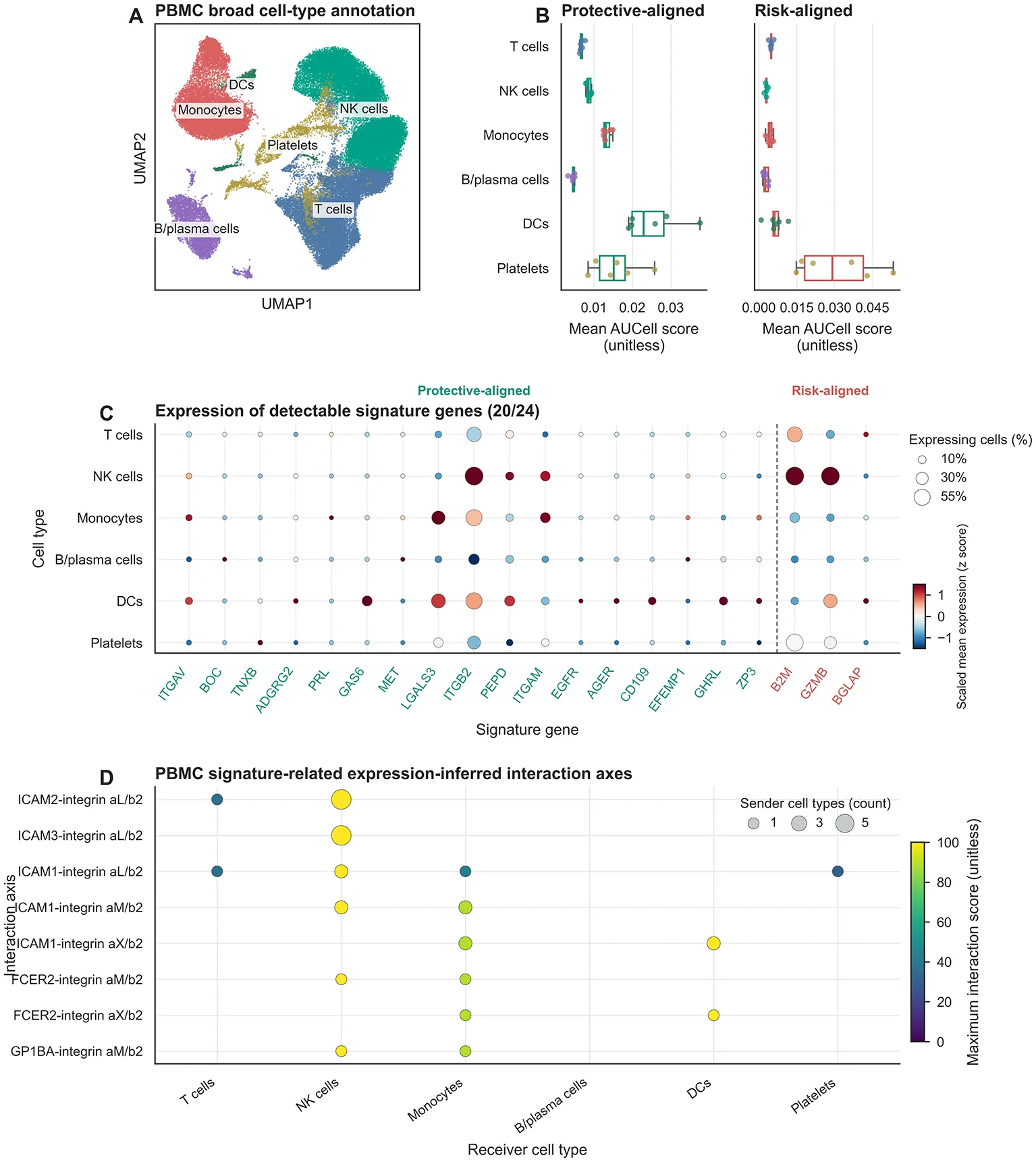
Cellular contextualization of the direction-compatible plasma proteomic signature in peripheral blood mononuclear cells. **(A)** UMAP of the PBMC scRNA-seq dataset. **(B)** Donor-level mean AUCell scores for the protective-aligned and risk-aligned signatures across major PBMC populations. **(C)** Expression of detectable signature genes across major PBMC populations. **(D)** Targeted CellPhoneDB analysis of signature-related ligand-receptor and adhesion interactions. AUCell, area under the curve-based gene set activity scoring; NK, natural killer; PBMC, peripheral blood mononuclear cell; scRNA-seq, single-cell RNA sequencing; UMAP, uniform manifold approximation and projection.

**Figure 5:**
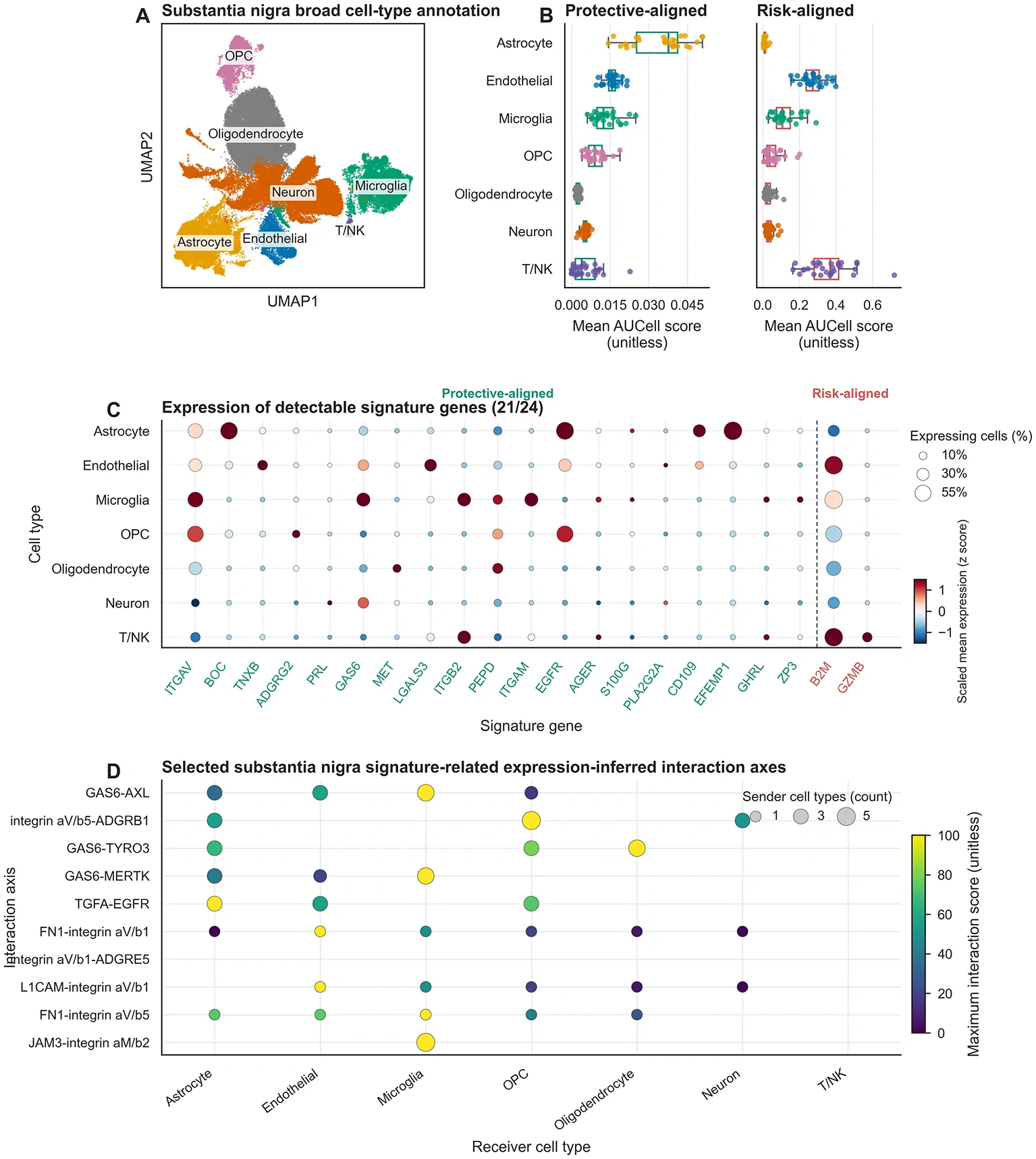
Cellular contextualization of the direction-compatible plasma proteomic signature in the human substantia nigra. **(A)** UMAP of the human substantia nigra snRNA-seq dataset. **(B)** AUCell-based activity scores of protective-aligned and risk-aligned proteomic signatures across major cell types. **(C)** Expression of detectable signature genes across major substantia nigra cell types. **(D)** Targeted CellPhoneDB analysis of signature-related ligand-receptor and adhesion interactions. AUCell, area under the curve-based gene set activity scoring; NK, natural killer; OPC, oligodendrocyte precursor cell; snRNA-seq, single-nucleus RNA sequencing; UMAP, uniform manifold approximation and projection.

Cellular contexts of the plasma proteomic signature were evaluated using single-cell and single-nucleus transcriptomic datasets. In the PBMC scRNA-seq dataset, major cell populations were annotated based on canonical marker genes (Figure 4A; Supplementary Figure 2). AUCell analysis showed that the protective-aligned signature exhibited higher AUCell scores in monocytes and natural killer (NK) cells, whereas the risk-aligned signature was highest in NK cells, followed by platelets and dendritic cells (Figure 4B). Gene expression analysis further showed that representative protective-aligned genes, including ITGAM and LGALS3, were expressed predominantly in monocyte- and dendritic-cell-related populations, whereas risk-aligned genes B2M and GZMB showed higher expression in NK/T-cell populations (Figure 4C). Targeted CellPhoneDB suggested predominantly protective-aligned, integrin-related interaction patterns involving ITGAM and ITGB2 (Figure 4D; Supplementary Tables 22–24). In the substantia nigra snRNA-seq dataset, cell annotation was similarly supported by canonical marker expression (Figure 5A; Supplementary Figure 3). Protective-aligned AUCell scores were relatively higher in astrocytes, with additional representation in endothelial cells and microglia, whereas risk-aligned scores were highest in T/NK cells (Figure 5B). ITGAM and ITGB2 showed immune- and microglia-associated expression, GAS6 showed broader expression across glial and neuronal populations, and EGFR showed higher expression in astrocyte- and oligodendrocyte precursor cell (OPC)-related populations; B2M and GZMB showed immune-associated expression (Figure 5C). CellPhoneDB analysis suggested integrin-, GAS6-, LGALS3-, and EGFR-related interaction patterns (Figure 5D; Supplementary Tables 22–24).

## 4 Discussion

In this prospective UK Biobank analysis of 123,655 participants with 796 incident hospital-recorded PD cases over a median follow-up of 13.7 years, higher FDS was associated with a modestly lower hospital-recorded PD incidence. The association was observed mainly among participants in the highest FDS quartile (*HR* 0.75, 95% *CI* 0.61–0.93), rather than as a strictly graded dose-response. The inverse direction was generally maintained across sensitivity analyses. However, estimates were attenuated in longer-lag analyses, and additional adjustment for coffee intake yielded a similar inverse point estimate but greater statistical uncertainty, indicating that prodromal dietary changes and residual lifestyle confounding cannot be excluded. Exploratory decomposition analyses identified no individual food source or flavonoid subclass that remained associated with incident hospital-recorded PD after multiple-testing correction. Proteomic prioritization and cellular contextualization in peripheral immune cells and the substantia nigra provided hypothesis-generating biological context rather than causal or mechanistic evidence. Overall, these findings support a small inverse observational association between FDS and incident hospital-recorded PD that requires confirmation in independent cohorts.

This pattern-level view, if correct, may help reconcile inconsistencies in the prior flavonoid literature. Earlier work suggested that certain flavonoid subclasses such as anthocyanins might be associated with a lower risk of PD (13), whereas other cohorts with long follow-up did not confirm this association (14), and a systematic review considered the available evidence suggestive rather than definitive (12). This may help reconcile the discrepancy above: unlike previous work that used single nutrients or total flavonoid intake as the exposure, the FDS emphasizes the combination of flavonoid-rich foods. In our data, no single food or subclass explained the association after FDR correction; in the same UK Biobank population, the diversity of flavonoid intake, independent of total quantity, was associated with lower risks of chronic and neurodegenerative diseases (24); and a National Health and Nutrition Examination Survey (NHANES) cross-sectional analysis showed a similar direction (25). Together, these observations are consistent with, but do not establish, a pattern-level interpretation in which the association reflects the combined profile of flavonoid-rich foods rather than any single component.

This distinction also delineates the potential incremental value of the FDS relative to established healthy-diet scores. Mediterranean and plant-based scores capture overall diet quality, and their associations with PD are driven largely by non-flavonoid components such as legumes, olive oil, and nuts (7, 8), which are not part of the FDS. Further adjustment for the Mediterranean diet adherence proxy produced little change in the FDS–PD estimate (HR approximately 0.92–0.93), suggesting that the association was not fully captured by this measure of overall diet quality; however, residual confounding and overlap between the dietary scores cannot be excluded. In magnitude, the HR of 0.75 for the highest FDS quartile is comparable to estimates of approximately 0.69–0.87 reported by prior Mediterranean and plant-based cohorts and meta-analyses (26–29), consistent in direction and magnitude but with a more focused exposure definition.

Baseline characteristics showed that participants with higher FDS were generally healthier; the inverse association persisted after adjustment for socioeconomic status, lifestyle, energy intake, multimorbidity, PD polygenic risk, and self-rated health, suggesting that it was not fully explained by these measured characteristics. Nonetheless, the healthy-user bias inherent to observational designs cannot be dismissed: individuals who favor flavonoid-rich foods tend to be healthier in many respects, and unmeasured or imperfectly measured factors may leave residual confounding (30). This was most evident for coffee. After adjustment for coffee intake, the point estimate was essentially unchanged, but the statistical evidence became borderline (*HR*, 0.93; 95% *CI*, 0.86–1.00; *p* = 0.056). Because coffee, a recognized factor inversely associated with PD risk (31), and tea overlap with the FDS and with broader beverage patterns, this may reflect residual confounding, exposure overlap, or overadjustment rather than the absence of an independent flavonoid-related association. Beyond confounding, the availability of dietary assessment was itself selective: compared with participants without a derived FDS, those with at least two valid WebQ assessments were more likely to be White, were less socioeconomically deprived, had lower prevalences of obesity and current smoking, and had a lower crude hospital-recorded PD incidence rate, indicating that WebQ participation may introduce healthy-volunteer selection (32). Overall, the association was directionally consistent across multiple sensitivity analyses, but its robustness is limited by this borderline effect, residual confounding, and selection bias.

Reverse causation also warrants consideration: some non-motor prodromal features (hyposmia, constipation, reduced appetite) can appear years before diagnosis and alter dietary behavior (33), and body weight declines in the years before diagnosis (34), so preclinical disease may modify prediagnostic diet and influence risk estimates. To reduce such bias, prior studies have applied longer lag periods; for example, the E3N cohort still observed an association between plant-based/Mediterranean diets (a broader exposure than the FDS) and lower PD risk after applying a 20-year lag with adjustment for prodromal symptoms (8). Constrained by follow-up duration, our lag analyses extended up to 10 years: the direction was maintained after excluding cases within the first 1–5 years, whereas estimates attenuated with wider confidence intervals in the 6–10-year windows. Thus, although extending the lag reduced the likelihood of reverse causation, an influence of preclinical disease on diet cannot be entirely excluded given the long prodromal phase.

From a biological standpoint, an inverse FDS-PD association is not implausible, since flavonoids and other polyphenols have antioxidant, anti-inflammatory, and cellular stress-modulating effects (35, 36) that overlap with processes implicated in PD, such as oxidative stress and neuroinflammation (37, 38). To explore molecular and cellular contexts potentially associated with this dietary pattern and PD risk relationship, rather than to establish mechanism, we performed an exploratory analysis integrating a direction-compatible plasma proteomic screen (24 proteins within an exploratory direction-compatible protein signature) with two publicly available single-cell references (PBMC and substantia nigra), the former for the peripheral-immune expression context and the latter for expression in glial, vascular, and immune cells of the PD-vulnerable region. Although formal enrichment was limited, the prioritized proteins showed reported involvement in neuroimmune and adhesion-related processes, broadly concordant with prior PD evidence. For example, GAS6-MerTK signaling within the TAM (TYRO3, AXL, and MERTK) receptor family participates in microglial clearance of α-synuclein (39), while AGER and LGALS3 have been linked to microglial responses to α-synuclein pathology (40, 41), ITGAM-, ITGAV-, and EGFR-related signals are also consistent with recent plasma proteomic studies of PD (42, 43), while B2M and GZMB may reflect cytotoxic lymphocyte and major histocompatibility complex class I (MHC-I)-related immune processes, respectively (44, 45). Because CellPhoneDB infers interactions from expression patterns rather than direct molecular binding, these findings should be interpreted as expression-inferred interaction contexts. Candidate-related interactions in PBMCs were predominantly represented by ITGAM/ITGB2-associated integrin axes, whereas the substantia nigra data additionally suggested GAS6-, LGALS3-, EGFR-, and integrin-related interactions (46). Experimental evidence that polyphenols can reduce α-synuclein aggregation, suppress neuroinflammatory responses, attenuate advanced glycation end product (AGE)–receptor for AGE (RAGE) signaling, and modulate adhesion-related pathways provides general biological plausibility (35, 36, 47–49), but does not establish that these processes explained the observed association. Overall, these exploratory findings provide biological context centered on neuroimmune and adhesion-related interfaces rather than evidence for a specific mechanism.

These findings should be interpreted as hypothesis-generating evidence for future PD risk research. Drawing on a large prospective UK Biobank cohort with long follow-up and repeated dietary assessment, the study adds population-level evidence that a flavonoid-rich dietary pattern was associated with lower PD risk, with no individual food or subclass remaining associated after multiple-testing correction. Although the association persisted after adjustment for Mediterranean-diet adherence, residual overlap with broader dietary patterns cannot be excluded; the FDS may therefore capture a complementary flavonoid-rich dietary dimension rather than simply overall diet quality. The integrated proteomic and single-cell analyses added complementary molecular and cellular context, pointing to neuroimmune and adhesion-related processes. Clinically, the per-1-SD association is small (*HR* 0.93) and not yet sufficient to guide dietary recommendations, but because flavonoid-rich foods are common, inexpensive, and readily modifiable, even a modest association, if causal, could still be relevant to population-level PD risk.

Several limitations should also be acknowledged. First, as an observational study, residual confounding and prodromal reverse causation cannot be excluded, as discussed above; in particular, PD-relevant exposures such as pesticides and other environmental or occupational hazards, head injury, medication and supplement use, and socioeconomic factors beyond TDI and education were unavailable or only coarsely captured, so the association should not be interpreted as causal. Second, the complete-case analysis was restricted to Oxford WebQ responders, who were healthier and less deprived, which may introduce selection bias. Third, although repeated WebQ assessments were averaged to improve the estimation of habitual dietary intake and showed moderate reproducibility, these analyses primarily support the reliability of FDS estimation rather than its absolute validity. Residual measurement error may remain because dietary intake was self-reported and objective biomarkers of flavonoid exposure were unavailable. Fourth, ascertaining PD from hospital and death-registry ICD-10 codes favors specificity but may under-ascertain milder or outpatient-managed cases. Outcome misclassification may have affected the estimates; although non-differential under-ascertainment would generally be expected to attenuate associations, differential ascertainment related to healthcare access or disease severity cannot be completely excluded. Fifth, because participants were predominantly of European ancestry and healthier than the general population, the findings may not generalize to populations with different flavonoid sources, dietary cultures, or PD diagnostic practices, and external validation is needed. Finally, the molecular and cellular analyses are exploratory: candidates were prioritized at a nominal threshold in a small proteomic subset, baseline diet and proteins cannot establish temporal ordering or causal relationships, and because the plasma and brain data derive from different populations and tissues, the single-nucleus analysis offers cellular context rather than evidence of direct dietary regulation of brain gene expression. Future studies incorporating external validation, longitudinal dietary and proteomic measurements, brain-relevant multi-omics data, and functional experiments are needed to further clarify the temporal relationship, causal direction, and biological basis of the association between flavonoid-rich dietary patterns and PD risk.

## 5 Conclusion

In conclusion, this study adds prospective, population-based evidence indicating that a flavonoid-rich dietary pattern is associated with a lower risk of incident hospital-recorded PD, and exploratory proteomic and single-cell analyses provide exploratory biological context centered on neuroimmune and adhesion-related processes. Although causality remains to be established, these findings support further investigation of flavonoid-rich dietary pattern as a potentially relevant lifestyle factor in PD risk research, to be examined in additional independent prospective cohorts and future functional validation studies.

## Data Availability

The UK Biobank data used in this study are available to eligible researchers upon application and approval by UK Biobank (https://www.ukbiobank.ac.uk/), this study was conducted under application No. 532248. The peripheral blood mononuclear cell single-cell RNA-sequencing dataset (GSE223138) and substantia nigra single-nucleus RNA-sequencing dataset (GSE243639) are publicly available in the Gene Expression Omnibus at https://www.ncbi.nlm.nih.gov/geo/query/acc.cgi?acc=GSE223138 and https://www.ncbi.nlm.nih.gov/geo/query/acc.cgi?acc=GSE243639, respectively. Derived data supporting the findings of this study are available from the corresponding author upon reasonable request, subject to UK Biobank data-access policies.
