# Supplementary material for: Flavonoid-rich dietary patterns and incident hospital-recorded Parkinson's disease: a prospective UK Biobank study
Source: Front Nutr. 2026 Aug 10;13:1893517. doi: 10.3389/fnut.2026.1893517 (PMC13500324; doi:10.3389/fnut.2026.1893517)
Supplement: Supplementary file 1 [file Image_1.pdf]

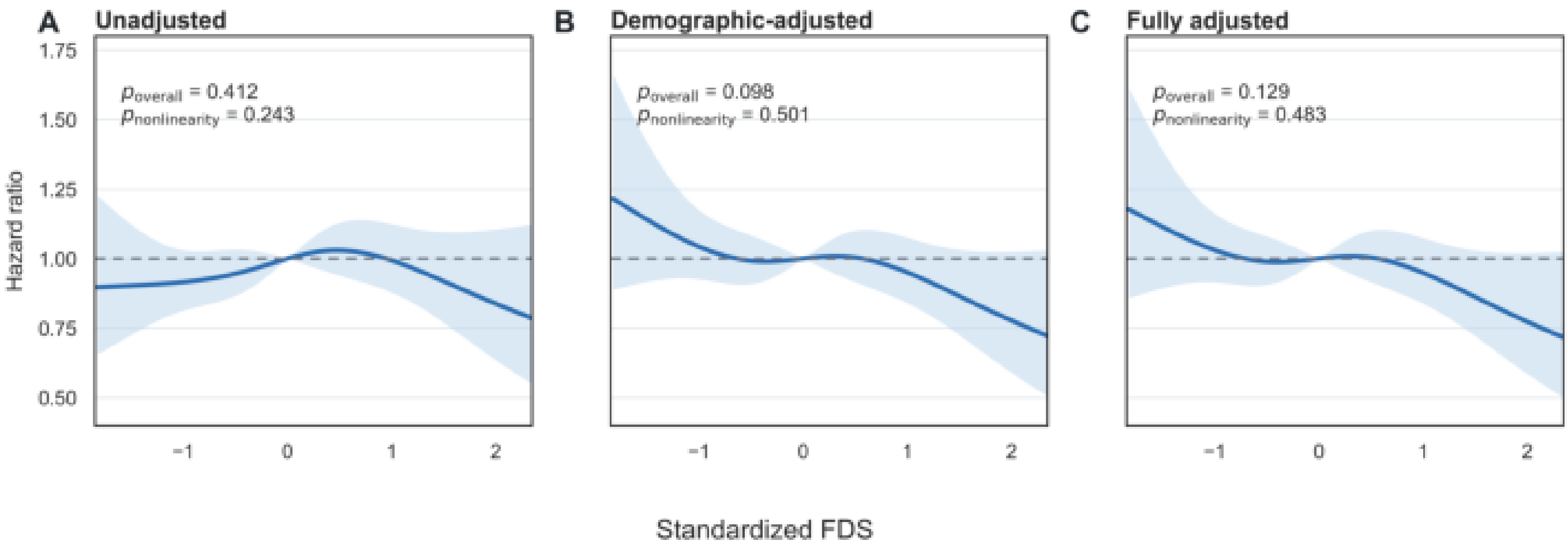

Supplementary Figure 1. Restricted cubic spline analyses of the association between standardized FDS and incident hospital-recorded PD across sequential Cox models. (A) Unadjusted model. (B) Demographic-adjusted model including age, sex, and ethnicity. (C) Fully adjusted model. Four knots were placed at the 5th, 35th, 65th, and 95th percentiles of the FDS distribution. The solid line represents the hazard ratio and the shaded area represents the 95% confidence interval, with standardized FDS = 0 as the reference. FDS, flavonoid diet score; PD, Parkinson's disease.

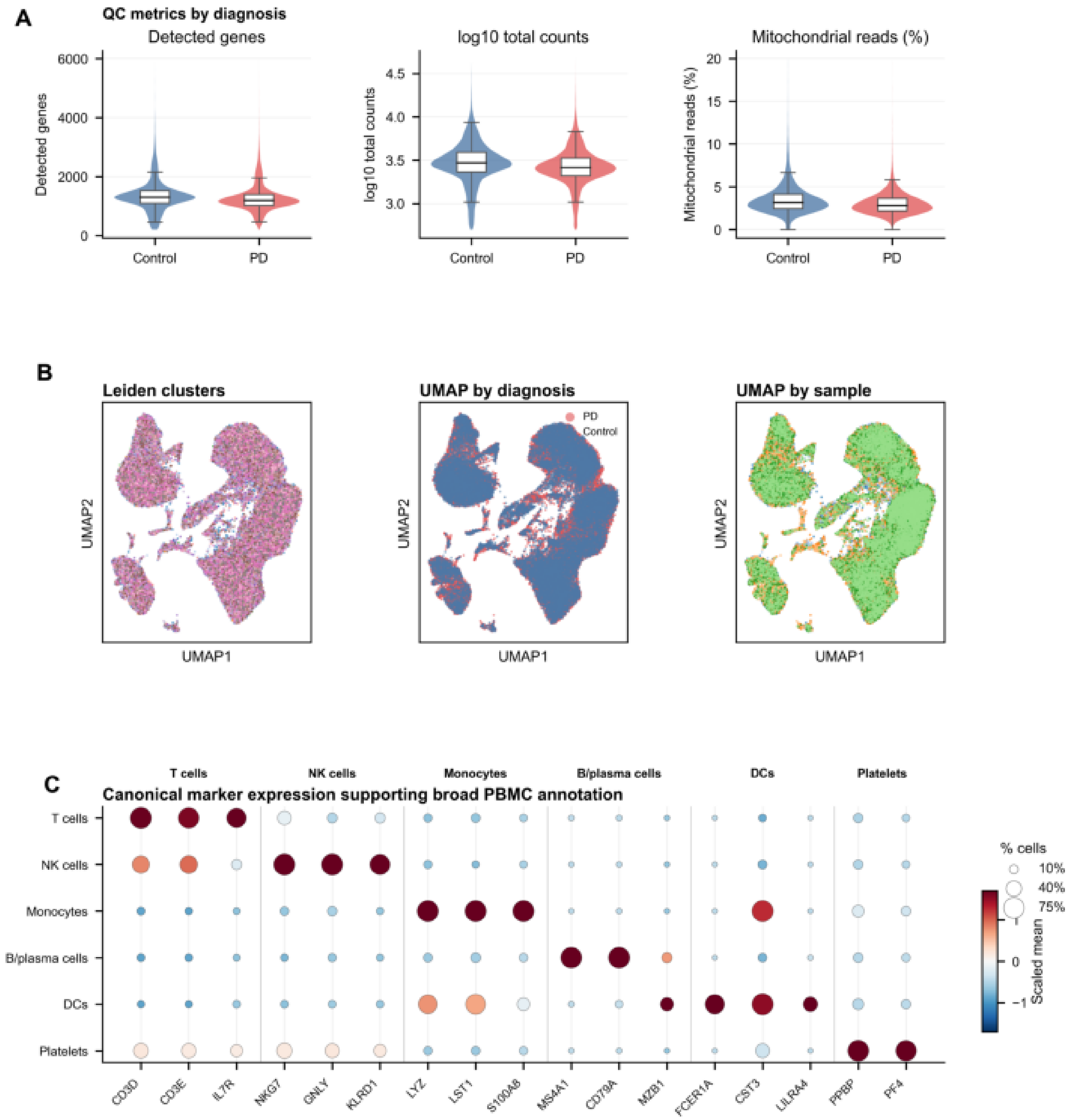

Supplementary Figure 2. Quality-control metrics, UMAP embeddings, and canonical marker-gene expression supporting broad PBMC cell-type annotation. (A) Distributions of detected genes, log10-transformed total counts, and mitochondrial reads according to diagnosis. (B) UMAP embeddings according to Leiden cluster, diagnosis, and sample. (C) Canonical marker-gene expression across broad PBMC populations. DCs, dendritic cells; PBMC, peripheral blood mononuclear cell; PD, Parkinson's disease; UMAP, uniform manifold approximation and projection.

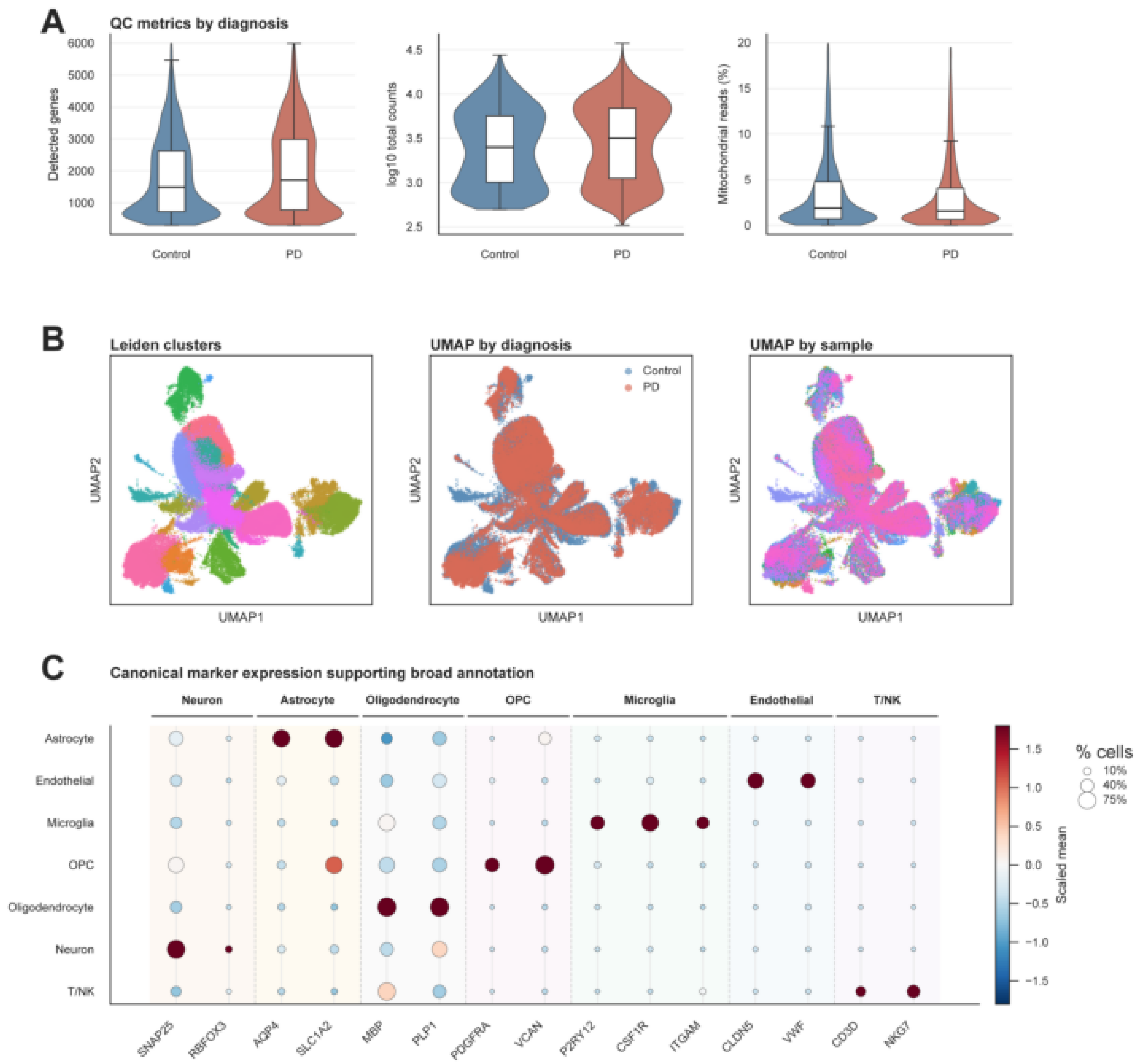

Supplementary Figure 3. Quality-control metrics, UMAP embeddings, and canonical marker-gene expression supporting broad substantia nigra single-nucleus annotation. (A) Distributions of detected genes, log10-transformed total counts, and mitochondrial reads according to diagnosis. (B) UMAP embeddings according to Leiden cluster, diagnosis, and sample. (C) Canonical marker-gene expression across broad cell populations. OPC, oligodendrocyte precursor cell; PD, Parkinson's disease; T/NK, T and natural killer cells; UMAP, uniform manifold approximation and projection.
